# Interleukin‐21 administration to aged mice rejuvenates their peripheral T‐cell pool by triggering *de novo* thymopoiesis

**DOI:** 10.1111/acel.12440

**Published:** 2016-01-13

**Authors:** E. Al‐Chami, A. Tormo, S. Pasquin, R. Kanjarawi, S. Ziouani, M. Rafei

**Affiliations:** ^1^ Department of PharmacologyUniversité de MontréalMontréalQCH3C 1J7Canada; ^2^ Université Paris‐Sud, Faculté de Pharmacie5 rue J.B. Clément92296Châtenay‐Malabry CedexFrance

**Keywords:** interleukin‐21, miR‐181a, T cells, thymic involution, tumor vaccine, signaling

## Abstract

The vaccination efficacy in the elderly is significantly reduced compared to younger populations due to thymic involution and age‐related intrinsic changes affecting their naïve T‐cell compartment. Interleukin (IL)‐21 was recently shown to display thymostimulatory properties. Therefore, we hypothesized that its administration to ageing hosts may improve T‐cell output and thus restore a competent peripheral T‐cell compartment. Indeed, an increase in the production of recent thymic emigrants (RTEs) attributable to intrathymic expansion of early thymic progenitors (ETPs), double‐negative (DN), and double‐positive (DP) thymocytes as well as thymic epithelial cell (TEC) was observed in recombinant (r)IL‐21‐treated aged mice. In sharp contrast, no alterations in the frequency of bone marrow (BM)‐derived progenitors were detected following rIL‐21 administration. Enhanced production of naïve T cells improved the T‐cell receptor (TCR) repertoire diversity and re‐established a pool of T cells exhibiting higher levels of miR‐181a and diminished amounts of the TCR‐inhibiting phosphatases SHP‐2 and DUSP5/6. As a result, stimulation of T cells derived from rIL‐21‐treated aged mice displayed enhanced activation of Lck, ZAP‐70, and ERK, which ultimately boosted their IL‐2 production, CD25 expression, and proliferation capabilities in comparison with T cells derived from control aged mice. Consequently, aged rIL‐21‐treated mice vaccinated using a tyrosinase‐related protein 2 (Trp2)‐derived peptide exhibited a substantial delay in B16 tumor growth and improved survival. The results of this study highlight the immunorestorative function of rIL‐21 paving its use as a strategy for the re‐establishment of effective immunity in the elderly.

## Introduction

In addition for being the key site of T lymphopoiesis in jawed vertebrates, the thymus maintains a competent peripheral T‐cell pool with a broad spectrum of TCR specificities (Lynch *et al*., 2009). It is, however, well established that immunity declines with ageing owing to two key factors impeding thymic function: a defect in the survival/proliferation ability of the prethymic hematopoietic progenitor pool coupled to the precocious loss of TECs (Boehm & Swann, 2013). These age‐related changes, collectively known as thymic involution, represent major driving forces for homeostatic expansion of preexisting peripheral T cells (Lynch *et al*., 2009). The net outcome culminates in TCR repertoire skewing with a noticeable increase in the number of effector/memory T cells (Zanni *et al*., 2003). Notably, a growing body of literature established that while the size of the peripheral T‐cell compartment remains unchanged throughout ageing, an increase in post‐thymic lifespan of T cells takes place consequently leading to the emergence of T‐cell intrinsic defects (Haynes & Swain, 2006; Maue *et al*., 2009; Tsukamoto *et al*., 2009). For instance, naïve CD4^+^ T cells derived from aged mice display defects in TCR threshold calibration, do not readily form immunological synapses, and have a marked reduction in the recruitment of TCR‐associated signaling molecules when compared to younger mice (Garcia & Miller, 1997, 2001, 2002; Tamir *et al*., 2000). Furthermore, increased expression of inhibitory receptors such as PD1, LAG3, 2B4, and CD160 were observed on the surface of ageing CD8^+^ T cells (Decman *et al*., 2012), while both IL‐2 secretion and proliferation potential are limited in naïve CD4^+^ and CD8^+^ T cells derived from aged mice (Eaton *et al*., 2004). Thus, stifled thymopoiesis combined to global qualitative changes affecting the ageing peripheral T‐cell pool limits the host's ability to mount effective responses against new antigenic challenges and accounts for the eroded immunity commonly observed in the elderly.

Primarily produced by activated CD4^+^ T cells, IL‐21 is a prominent member of the common γ‐chain family of cytokines (Spolski & Leonard, 2014). Besides its wide‐ranging effects on immune cells, IL‐21 overexpression *in vivo* triggers expansion of BM‐derived progenitors (Ozaki *et al*., 2006). Furthermore, we recently reported a novel mitogenic function for IL‐21 on peptide‐mediated TCR‐engaged DP thymocytes using a newly developed *in vitro* coculture system designed for T‐cell differentiation (Rafei *et al*., 2013b). Likewise, rIL‐21 administration to mice with glucocorticoid‐induced thymic atrophy dramatically accelerates thymic function recovery by stimulating the proliferation of ETPs, DN, and positively selected DP thymocytes (Rafei *et al*., 2013a). Such unprecedented thymopoiesis‐supporting function suggests that rIL‐21 is indeed a promising therapeutic tool endowed with the capacity of improving T‐cell output in aged hosts owing to the expression of the IL‐21 receptor (IL‐21R) on both BM and thymic progenitors (Ozaki *et al*., 2006; Rafei *et al*., 2013a,b). We wished therefore to scrutinize whether rIL‐21 administration to aged mice can rejuvenate their T‐cell immunity by targeting *de novo* thymopoiesis as a mean to enhance their antitumoral response following vaccination.

## Results

### Administration of rIL‐21 enhances thymopoiesis in aged mice

**Figure 1:**
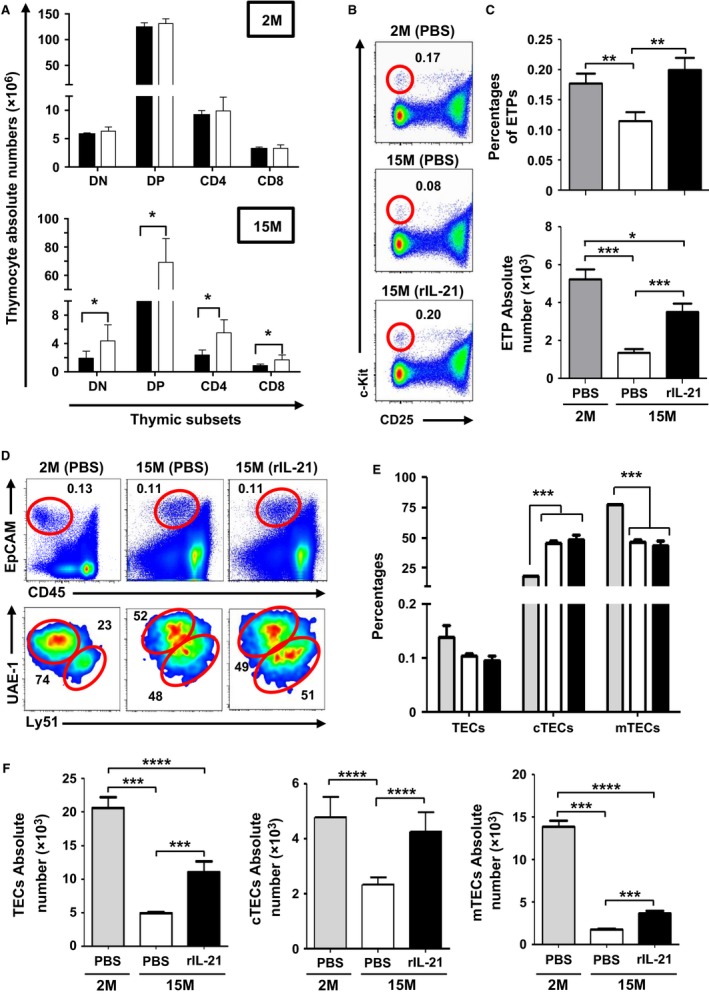
Administration of rIL‐21 promotes *de novo* thymopoiesis in aged but not young mice. (A) Counts of thymocyte subpopulations. Groups are displayed as PBS (■) and IL‐21 (□). (B) Representative flow cytometry analysis of ETPs. (C) Absolute count of ETPs. (D, E) Percentages of total, cTECs, and mTECs. (F) Absolute counts of total, cTECs, and mTECs in comparison with PBS‐treated aged mice. For panels C, E, and F, groups are displayed as: 2M (PBS 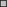), 15M (PBS □), or 15M (rIL‐21 ■). All data are representative of three independent experiments (*n* = 5/group with *P < 0.05, **P < 0.01, ***P < 0.001, and ****P < 0.0001).

To ensure maximal thymopoiesis‐stimulating effects *in vivo*, we first conducted a dose–response study in young (2 months—2M) versus old (15 months—15M) RAG2p‐GFP mice by intraperitoneally (IP) administering rIL‐21 (Fig. S1A). The use of the RAG2p‐GFP model allows to easily assess *de novo* thymopoiesis as expression of the GFP transgene, marking newly developed T cells, is controlled by the *Rag2* promoter activity (Monroe *et al*., 1999; Yu *et al*., 1999; Rafei *et al*., 2011). Thymic analysis 1 week following the last injection revealed a progressive increase in total thymocyte count in 15M but not 2M‐old rIL‐21‐treated mice with an optimal response rate achieved at a dose of 50 μg kg^−1^ (Fig. S1B). Similarly, only aged mice receiving rIL‐21 exhibited an increase in the counts of all thymic subsets (DN, DP and single positive) (Fig. 1A) including ETPs (Fig. 1B,C). Even though the percentage of GFP^+^ thymocytes remained unchanged in all studied groups (Fig. S1C), the increased thymic count observed in the rIL‐21‐treated aged mice was sustained over a 3‐week period postcytokine administration (Fig. S1D). To determine whether rIL‐21‐enhanced thymopoiesis involves the expansion of BM progenitors, which could have increased their migration rate to the thymus, we next monitored the frequency of LSK cells (Lin^−^ Sca1^+^ c‐Kit^+^) and its subpopulations including the long‐term (LT; Lin^−^ Sca1^+^ c‐Kit^+^ CD34^−^ CD135^−^) and short‐term (ST; Lin^−^ Sca1^+^ c‐Kit^+^ CD34^+^ CD135^−^) hematopoietic stem cells (HSCs), as well as multipotent progenitors (MPPs; Lin^−^ Sca1^+^ c‐Kit^+^ CD34^+^ CD135^+^) following rIL‐21 treatment. Despite IL‐21R expression on the surface of wild‐type (WT) LT, ST‐HSCs, and MPPs (Fig. S2A), the overall proportion of LSK cells (Fig. S2B) or its subpopulations (Fig. S2C) was not affected by rIL‐21 administration. Likewise, no increase in the number of LSK subpopulations (Fig. S2D) nor in the more differentiated common lymphoid progenitor (CLP; Lin^−^ IL‐7R^+^ Sca1^+^ c‐Kit^+^) population (Fig. S2E) was observed. Furthermore, only *in vitro* cultured young LSK cells proliferated when cultured with rIL‐21 (Fig. S2F) clearly suggesting a defective response in ageing BM.

We recently reported that TECs are devoid of IL‐21R (Rafei *et al*., 2013a). Therefore, we presumed that the thymic effects observed following rIL‐21 infusion strictly affect hematopoietic cells. Indeed, when given to aged mice, rIL‐21 does not fluctuate the frequency of total TECs (EpCAM^+^ CD45^−^), nor it did affect the ratio of cortical (c)TEC (EpCAM^+^ CD45^−^ UAE‐1^−^ Ly51^+^) relative to medullary (m)TEC (EpCAM^+^ CD45^−^ UAE‐1^+^ Ly51^−/lo^) populations (Fig. 1D,E). Conversely, absolute counts analysis showed marked improvements in the stromal compartment as total, cTEC, and mTEC populations were significantly higher in rIL‐21‐treated aged mice compared to the PBS group (Fig. 1F). Higher production of IL‐7/thymus production was also noticed in the rIL‐21‐treated aged mice (Fig. S1E). These data suggest that rIL‐21 administration is beneficial to aged mice by directly targeting thymopoiesis *in situ* without triggering the expansion of BM‐derived LSK cells.

### Physiological levels of RTE are restored in aged mice following rIL‐21 treatment

**Figure 2:**
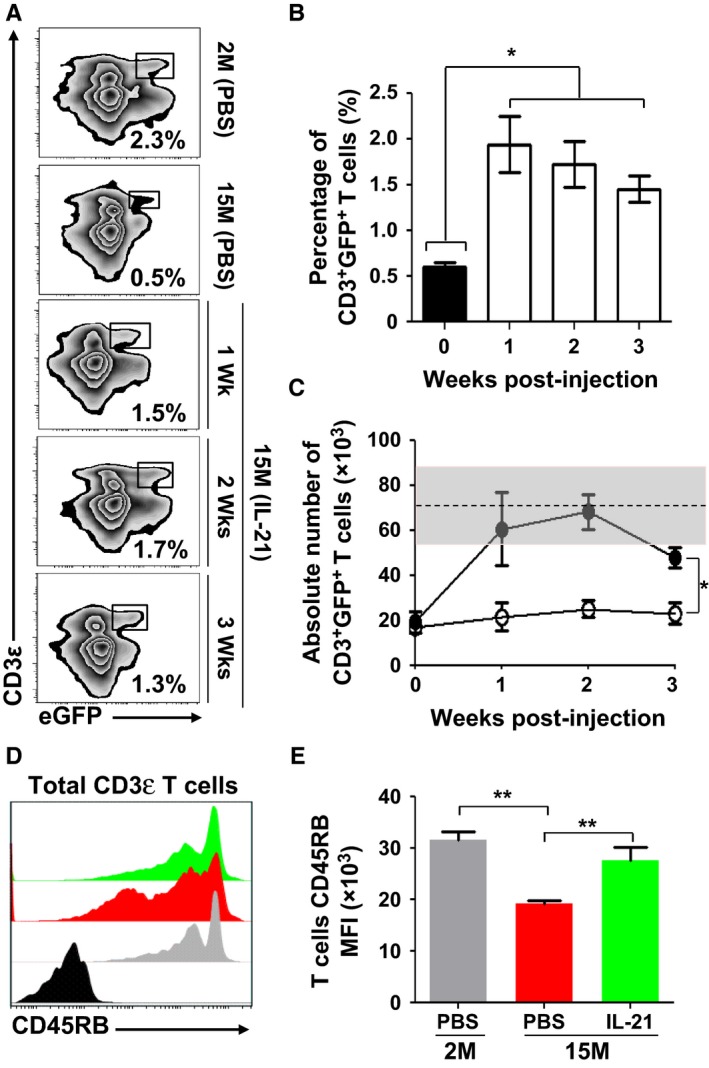
Increased levels of circulating RTEs in rIL‐21‐treated aged mice. (A) Representative flow cytometry analysis of RTEs in peripheral blood of young (2M) or aged (15M) mice 1, 2, or 3 weeks post‐rIL‐21 administration. Young mice treated with PBS were used as comparative positive controls. (B, C) Analysis of overall percentages (B) and counts (C) of RTEs in the weeks following rIL‐21 administration to aged mice. The black histogram represents the 2M PBS‐treated mice. The gray zone in (C) represents the RTE level calculated using 2M young mice (*n* = 10) and displayed as the average RTE number ± two SD. Black circles in (C) represent rIL‐21‐treated mice. (D) Representative flow cytometry analysis of CD45RB on the surface of all CD3^+^ T cells derived from 2M (PBS 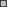), 15M (PBS 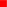), and 15M (rIL‐21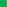). CD45RB isotype is displayed in black. (E) Compiled MFIs for CD45RB expression in treated mice. All data are representative of three independent experiments (*n* = 5/group with *P < 0.05 and **P < 0.01).

To interrogate the functional relevance of rIL‐21‐enhanced thymopoiesis on the peripheral T‐cell pool of aged RAG2p‐GFP mice, we next assessed the percentage of circulating RTEs mirrored by the level of peripheral GFP^+^ CD3ε^+^ T cells. In contrast to 2M‐old animals, where RTEs represent roughly 2.3% of total circulating lymphocytes, lower percentages (~0.5%) are detected in the peripheral blood of 15M PBS‐treated aged mice (Fig. 2A). Following rIL‐21 treatment, the percentage of GFP^+^ CD3ε^+^ T cells reached a range of 1.3–1.7% over a period of 3‐week postcytokine treatment (Fig. 2A,B) with absolute numbers attaining physiological levels according to RTE counts calculated using blood derived from young mice (Fig. 2C).

With increased encountered antigens and declined RTE levels, qualitative changes in the phenotype of peripheral T‐cell composition occur with ageing (Boursalian *et al*., 2004). More specifically, the expression of various cell surface makers including the glycoprotein CD45RB is downregulated as T cells become activated and progress from a naïve to a memory phenotype (Tough & Sprent, 1994). We therefore hypothesized that enhancing RTE generation in aged mice would increase the overall expression pattern of CD45RB on peripheral T‐cell pool and found that it was indeed the case in aged mice treated with rIL‐21 as depicted by histogram overlaps (Fig. 2D) and compiled mean fluorescent intensities (MFIs) (Fig. 2E). We therefore conclude that rIL‐21 treatment enhances the *de novo* generation of RTEs, which incorporate the peripheral T‐cell pool of aged mice.

### The nature of ageing T‐cell pool is greatly affected by rIL‐21 administration

**Figure 3:**
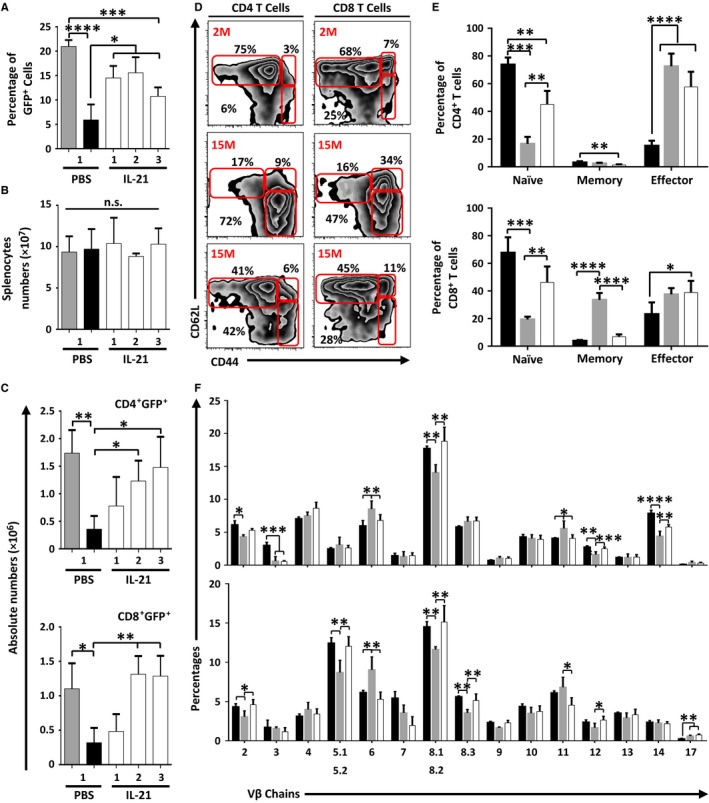
Aged mice treated with rIL‐21 display increased proportion of naïve T cells with enhanced TCR diversity. (A) Percentages of GFP ^+^ events in the spleen of treated mice. (B) Splenocyte counts in all experimental groups. (C) Absolute counts of CD4^+^ GFP ^+^ and CD8^+^ GFP ^+^ T cells in treated mice. (D) A representative flow cytometry analysis of naïve (CD62L^hi^ CD44^lo^), memory (CD62L^hi^ CD44^hi^), and effector (CD62L^lo^ CD44^hi^) T cells in all experimental groups. (E) Compiled percentages of all three subpopulations in CD4^+^ (top panel) and CD8^+^ (lower panel) T cells. (F) Flow cytometry analysis of 15 TCRVβ chains using peripheral CD4^+^ (top panel) or CD8^+^ (lower panel) T cells. For panels A–C, groups are displayed as 2M (PBS 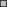), 15M (PBS ■), and 15M (rIL‐21 □). For panels E and F, groups are displayed as 2M (PBS ■), 15M (PBS 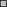), and 15M (rIL‐21 □). All data are representative of three independent experiments (*n* = 5/group with *P < 0.05, **P < 0.01, ***P < 0.001, and ****P < 0.0001).

Following thymic egress, RTEs continue their maturation in the periphery to eventually become fully competent mature naïve T cells (Boursalian *et al*., 2004). To do so, they require access to secondary lymphoid organs (SLO) as a mean to encounter other cell types and cytokines required for their maturation (Houston *et al*., 2008). Although the percentage of GFP^+^ T cells increased significantly in the spleen of rIL‐21‐treated aged mice in the 3 weeks following cytokine treatment (Fig. 3A), the overall number of splenocytes remained steady (Fig. 3B). Further in‐depth analysis revealed a progressive time‐dependent increase in the absolute counts of GFP^+^ CD4^+^ and GFP^+^ CD8^+^ T cells in the spleens of rIL‐21‐treated aged mice (Fig. 3C) suggesting that SLO‐resident aged T cells were displaced by newly migrating RTEs. We next examined by flow cytometry the differentiation stages of spleen‐derived CD4^+^ and CD8^+^ T cells and detected increased abundance of T cells with a naïve phenotype (CD44^lo^CD62L^hi^) in IL‐21‐treated aged mice (Fig. 3D,E) bolstering the notion of rIL‐21‐mediated enhanced T‐cell output.

Given that thymic involution compromises the TCR repertoire (Yager *et al*., 2008; Ahmed *et al*., 2009), we continued our analysis by investigating the effect of rIL‐21 administration on TCR diversity. To address this question, the expression profile of 15 TCRVβ‐chains was analyzed on the surface of spleen‐derived CD4^+^ and CD8^+^ T cells collected from treated mice. Although no changes occurred in the TCRVβ‐chains of young mice treated with PBS or rIL‐21 (Fig. S3), significant improvements were observed in the proportion of Vβ2‐, 6‐, 8.1/8.2‐, 11‐, 12‐, and 14‐expressing CD4^+^ T cells as well as Vβ2‐, 5.1/5.2‐, 6‐, 8.1/8.2‐, 8.3‐, 11‐, and 12‐expressing CD8^+^ T cells following rIL‐21 administration to ageing mice (Fig. 3F). These results indicate that rIL‐21‐mediated *de novo* RTE generation is associated with major qualitative changes in the peripheral T‐cell pool of aged mice including improved TCR diversity.

### Characterizing the biochemical responses of T cells

**Figure 4:**
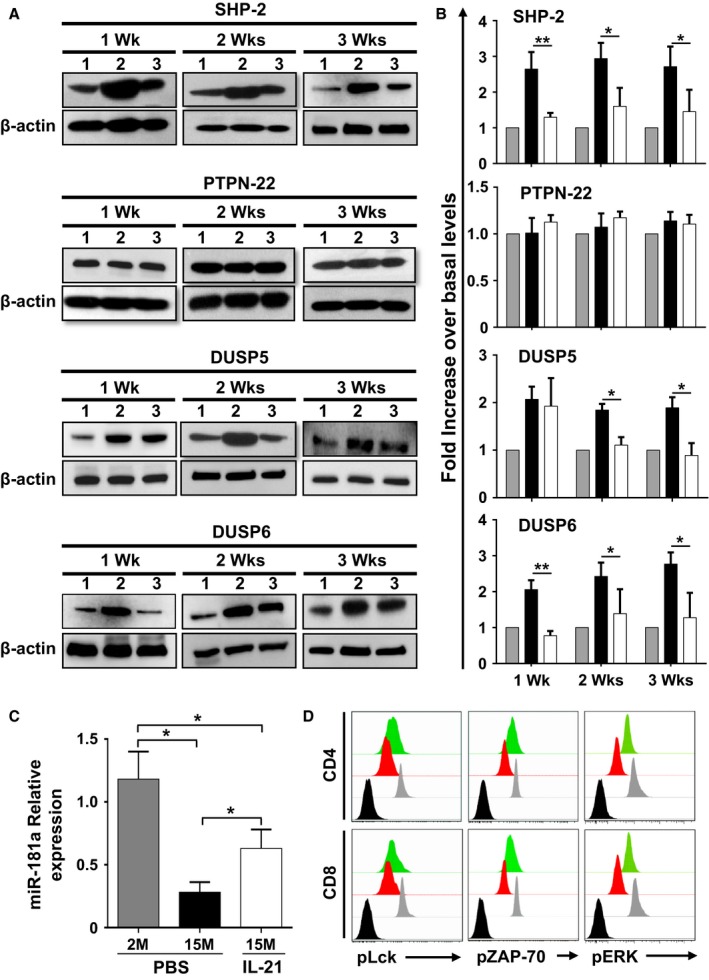
The peripheral pool of T cells in rIL‐21‐treated aged mice displays improved TCR signaling responses. (A) Representative Western blot analyses of phosphatases at 1, 2, or 3 weeks post‐treatments of 2M PBS (1), 15M PBS (2), and 15M rIL‐21 (3) aged mice. β‐actin was used as internal loading control. (B) Compiled densitometry analysis of phosphatase expression levels. The displayed groups are as follows: 2M (PBS 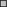), 15M (PBS ■), and 15M (rIL‐21 □) aged mice. C) qPCR analysis of miR‐181a in freshly isolated T cells from 2M (PBS 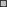), 15M (PBS ■), and 15M (rIL‐21 □) treated mice. D) Representative intracellular flow cytometry staining of pLck, pZAP‐70, and pERK in 2M (PBS 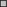), 15M (PBS 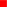), and 15M (rIL‐21 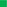) aged mice. Nonstimulated T cells derived from young 2M mice are displayed by black histograms. All data are representative of three independent experiments (*n* = 5/group with *P < 0.05, and **P < 0.01).

Thymic involution cannot solely account for impaired immune responses as additional T‐cell intrinsic defects appear in ageing naïve T cells due to prolonged post‐thymic lifespan (Haynes & Swain, 2006; Maue *et al*., 2009; Tsukamoto *et al*., 2009). More specifically, TCR activation is blunted with ageing due to increased cytoplasmic concentration of phosphatases known for inhibiting TCR signaling (Li *et al*., 2012). Given such fluctuation in TCR threshold calibration, we next explored whether the previously observed changes in the nature of the peripheral T‐cell pool induced by rIL‐21 affect the expression levels of the TCR‐targeting phosphatases SHP‐2, PTPN‐22, and DUSP5/6. Delineation of phosphatase levels by Western blotting (Fig. 4A) and densitometry‐based quantification (Fig. 4B) revealed diminished SHP‐2 and DUSP5/6 levels in T cells derived from rIL‐21‐treated aged mice at all tested time points. Only PTPN‐22 remained unchanged in all groups throughout treatments (Fig. 4A,B). Previous studies conducted in mice demonstrated that SHP‐2 and DUSP5/6 were among several phosphatases controlled by miR‐181a, a ~22nt microRNA molecule capable of repressing the translation of over 40 phosphatases (Li *et al*., 2007). Consistently, the progressive decline in miR‐181a levels observed in ageing human naïve CD4^+^ T cells dovetails the decreased T‐cell responsiveness following TCR stimulation (Li *et al*., 2012). To examine the possibility that rIL‐21 could have reversed such defect through enhanced generation of RTEs expressing normal levels of miR‐181a, quantitative (q)PCR studies were conducted using T cells sorted from spleens of treated mice. Our analysis revealed a two‐ to threefold increase in miR‐181a levels in T cells derived from rIL‐21‐treated aged mice (Fig. 4C), which is consistent with the diminished SHP‐2 and DUSP5/6 levels observed earlier (Fig. 4A,B). We next tested the responses of T cells derived from treated mice by probing early signaling events triggered by TCR stimulation. Although not efficient as younger lymphocytes, CD4^+^ or CD8^+^ T cells derived from rIL‐21‐treated aged mice displayed higher Lck, ZAP‐70, and ERK phosphorylation compared to control PBS aged mice as shown by phospho‐flow analysis (Fig. 4D). Taken collectively, these data indicate that enhanced TCR responses in the pool of T cells derived from rIL‐21‐treated aged mice are attributable to the increase in the proportion of naïve T cells displaying lower SHP‐2 and DUSP5/6 phosphatase levels owing to improved miR‐181a expression.

### rIL‐21 administration improves the biological responses of T cells derived from aged mice

**Figure 5:**
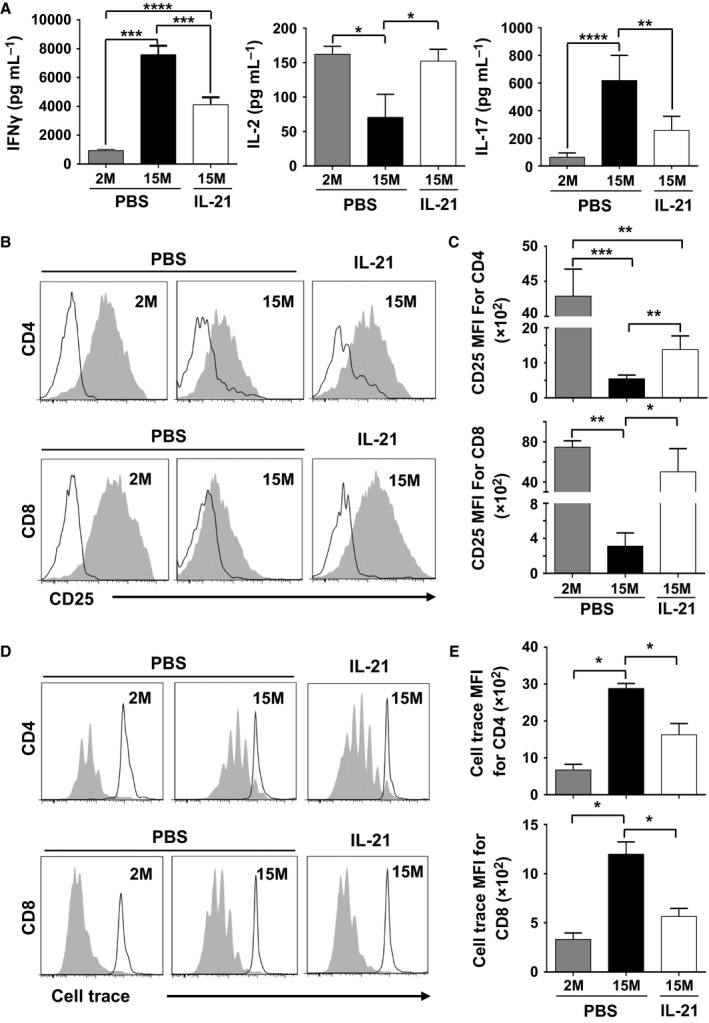
Enhanced biological responses of peripheral T‐cell pool derived from rIL‐21‐treated aged mice. (A) Cytokine quantification from stimulated CD3^+^ T cells isolated from the spleen of treated mice. (B) Representative flow cytometry analysis of CD25 cell surface expression on CD4^+^ (top panels) and CD8^+^ (lower panels) T cells. (C) Compiled MFIs for CD25 expression on CD4^+^ and CD8^+^ T cells. (D) Representative flow cytometry analysis of cell trace dilution following TCR stimulation of CD4^+^ (top panels) and CD8^+^ (lower panels) T cells derived from treated animals. (E) Compiled MFIs for cell trace dilution of CD4^+^ and CD8^+^ T cells following stimulation. All data are representative of three independent experiments (*n* = 5/group with *P < 0.05, **P < 0.01, ***P < 0.001, and ****P < 0.0001).

The dramatic reduction in naïve T‐cell output coupled to the relative increase in the proportion of effector and central memory T cells in aged mice negatively impact T‐cell responses to neo‐antigens (Yager *et al*., 2008). Repeated observations revealed that beside scarcity of T‐cell precursors in the pre‐immune repertoire, IL‐2 production, cell surface expression of CD25, and T‐cell proliferation are all greatly reduced in ageing hosts (Haynes & Swain, 2006; Maue *et al*., 2009; Tsukamoto *et al*., 2009). Elevated secretion levels of pro‐inflammatory cytokines such as interferon (IFN)γ caused by the accumulation of CD44^hi^ T cells were also reported to play a role in accelerating age‐related immunosenescence (Zhang *et al*., 2002; Decman *et al*., 2012). The observations made so far led us to speculate that the beneficial effect of rIL‐21 on thymopoiesis in aged mice should improve the effector function of their rejuvenated T‐cell pool. To test this hypothesis, spleen‐derived CD3ε^+^ T cells were isolated 3 weeks following the last cytokine treatment (to ensure that they had completed their necessary post‐thymic maturation) and stimulated using CD3‐CD28 beads (Berkley *et al*., 2013). Not only were IFNγ and IL‐2 secretion profiles by TCR‐stimulated T cells derived from rIL‐21‐treated mice comparable to younger animals, but a striking decrease in IL‐17 production was observed as well (Fig. 5A). In addition, the intensity of CD25 cell surface expression on spleen‐purified TCR‐stimulated CD4^+^ or CD8^+^ T cells and their proliferation rates were comparable between young and rIL‐21‐treated aged mice as determined by flow cytometry (Fig. 5B,D) and compiled MFIs data (Fig. 5C,E). To gain further insights, we quantified by qPCR the expression level of several genes known to influence the differentiation and/or effector function of T cells. Although no difference was observed in *Nfat*,* Ap‐1,* and *Gata3* expression in all tested groups, a decrease in *T‐bet* and *RORC* (encoding for RORγt) transcript levels was detected in T cells derived from rIL‐21‐treated aged mice compared to aged PBS control mice (Fig. S4). *Foxp3* expression level, on the other hand, was equivalent between PBS‐ and rIL‐21‐treated aged mice but significantly higher than younger mice (Fig. S4). These qPCR results are consistent with the observed decrease in IFNγ (*T‐bet*) and IL‐17 (*RORC*) secretion levels and are particularly interesting as several investigations have associated IL‐21 to autoimmune diseases (Korn *et al*., 2007; Peluso *et al*., 2007; Attridge *et al*., 2012). Further analyses conducted on rIL‐21‐treated aged mice showed no unusual signs of inflammation or autoimmunity (Fig. S5). More specifically, we found (i) that the spleen size and weight of PBS‐ or rIL‐21‐treated aged mice were similar to younger mice (Fig. S5A), (ii) that the total serum IgG levels were lower in young mice but comparable in both PBS‐ and rIL‐21‐treated animals indicating an age‐related factor at play independent of rIL‐21 treatment (Fig. S5B), and (iii) no immune infiltrates in lungs, liver, or kidneys of aged mice receiving rIL‐21 (Fig. S5C). In agreement with previous studies reporting accumulation of CD25^+^ CD4^+^ T_regs_ in the periphery and SLO of ageing mice and humans (Lages *et al*., 2008), we detected a significantly higher percentage and absolute number of spleen‐derived T_regs_ in both PBS‐ and rIL‐21‐treated aged mice when compared to their younger counterparts (Fig. S5D,E). Globally, these findings indicate that rIL‐21‐mediated boosting of T‐cell output in aged mice corrects the T‐cell dysfunctionalities commonly seen with ageing without inducing signs of autoimmunity.

### Enhanced vaccine‐elicited antitumoral response in rIL‐21‐treated aged mice

**Figure 6:**
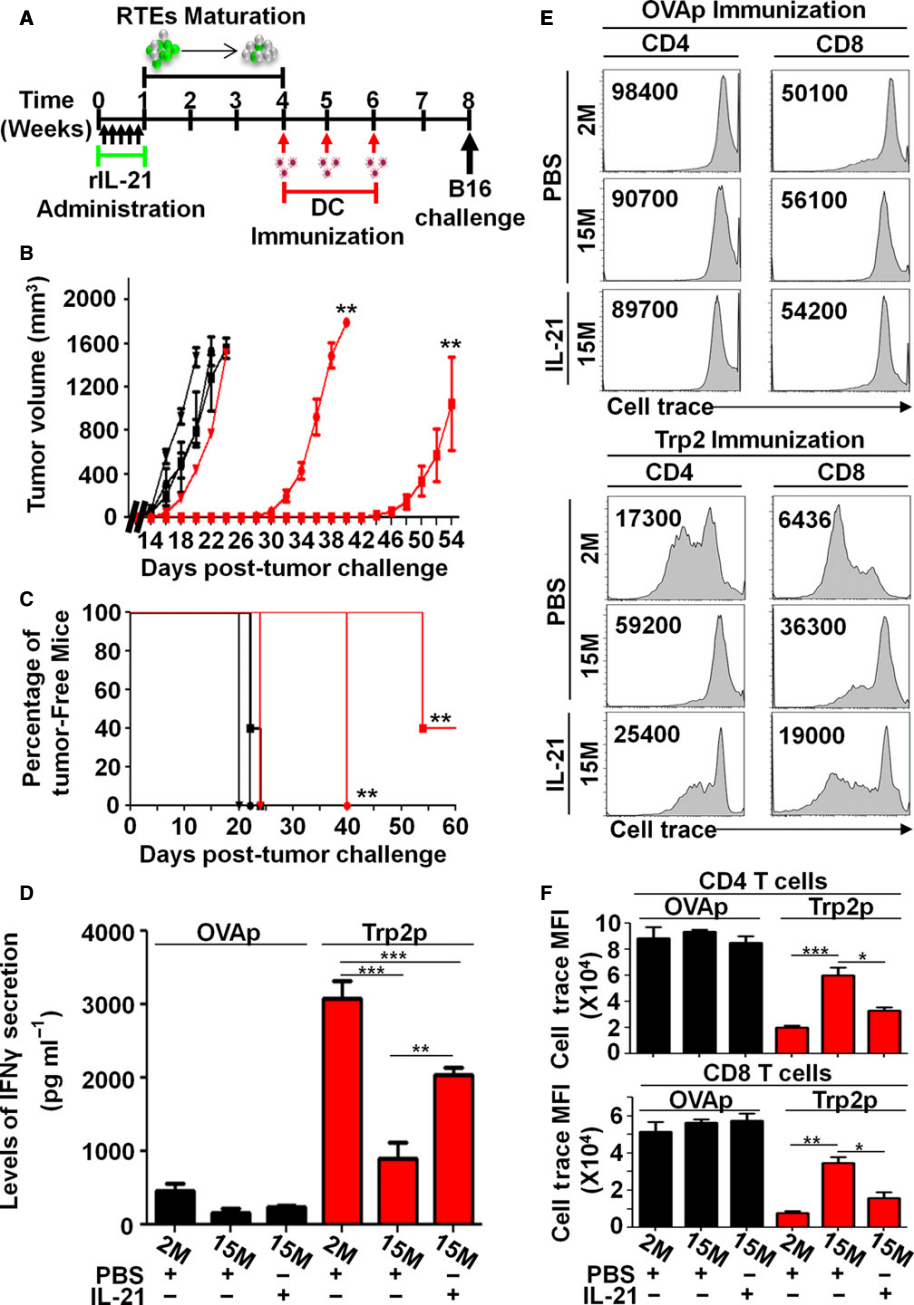
T‐cell rejuvenation of aged mice enhances their antitumoral immunity. (A) Timeline used for vaccination. (B, C) Tumor volumes and percentage of tumor‐free mice post‐B16 tumor cell challenge in OVAp‐DC‐vaccinated 2M (PBS ■), 15M (PBS ▼), and 15M (rIL‐21 ●); or Trp2p‐DC‐vaccinated 2M (PBS 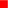), 15M (PBS 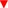) and 15M (rIL‐21 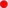). (D) IFNγ quantification following *in vitro* Trp2p stimulation of T cells derived from vaccinated animals. (E) Representative flow cytometry analysis of cell trace dilution in CD4^+^ and CD8^+^ T cells derived from vaccinated animals following *in vitro* Trp2 stimulation. (F) Compiled MFIs for cell trace dilutions in CD4^+^ (top panel) and CD8^+^ (lower panel) T cells following *in vitro* Trp2 stimulation. OVAp (SIINFEKL) and Trp2p (SVYDFFVWL). All data are representative of three independent experiments (*n* = 5/group with *P < 0.05, **P < 0.01, and ***P < 0.001).

To evaluate the effectiveness of our T‐cell rejuvenation therapy, a proof‐of‐concept vaccination study was conducted using an experimental melanoma antigen (Parkhurst *et al*., 1998). Briefly, aged mice were first treated with PBS or rIL‐21 according to our established protocol, then left for 3 weeks to allow for RTE maturation (Fig. 6A). All mice were then vaccinated weekly (for a total of three injections) using *in vitro* generated BM‐derived mature DCs (Fig. S6) pulsed with SIINFEKL (a control epitope derived from chicken ovalbumin) or SVYDFFVWL peptides (the experimental epitope derived from the melanoma differentiation antigen Trp2). Two weeks following the last DC boost, mice were challenged subcutaneously (SC) with syngeneic B16 tumor cells and monitored thereafter for tumor growth and survival. Although all SIINFEKL‐vaccinated mice died within the same time frame (day 20–24) with no statistically significant difference between young, PBS‐, or rIL‐21‐treated mice, a slower tumor growth (Fig. 6B) and a significant delay in survival (Fig. 6C) were observed in the Trp2‐vaccinated rIL‐21‐treated aged mice in comparison with the Trp2‐vaccinated PBS control group (survived up to 40 versus 22 days, respectively). We then retrieved the spleens of mice from each group and analyzed their IFNγ production level and proliferation following Trp2 restimulation *in vitro*. All Trp2‐vaccinated animals responded to peptide restimulation with a significant increase in IFNγ response achieved in rIL‐21‐treated aged mice compared to control PBS aged mice (Fig. 6D). Similarly, enhanced proliferation responses were observed in the rIL‐21‐treated aged mice as evaluated by cell trace dilution overtime by flow cytometry (Fig. 6E) and compiled MFIs data (Fig. 6F). In sum, these data clearly show that rIL‐21 preconditioning of aged mice leads to marked improvements in the control of B16 tumor growth following Trp2 vaccination.

## Discussion

Thymic involution deprives aged hosts from a competent immune system capable of effectively responding to vaccination and invading pathogens (Boehm & Swann, 2013). Although some aspects of age‐related decline in T‐cell responses reflect systemic changes, others are due to cell intrinsic defects (Haynes & Swain, 2006; Maue *et al*., 2009; Tsukamoto *et al*., 2009). We show in this report that administration of rIL‐21 enhances thymopoiesis in aged mice through expansion of both the stromal and responsive thymocytes compartments without the induction of any apparent pathology in peripheral organs. Consequently, a competent peripheral lymphoid pool containing larger proportion of naïve CD4^+^ and CD8^+^ T cells (CD62L^hi^CD44^lo^) displaying potent effector functions in response to TCR stimulation was restored. This increase in the availability and potency of naïve T cells augmented the responsiveness of aged mice to Trp2 vaccination and accounts for their improved survival in response to B16 tumor challenge (Fig. S7).

T lymphopoiesis remains functional at older age albeit to a limited extent (Hale *et al*., 2006). As the thymus lacks self‐renewing progenitors, it heavily relies on sustained seeding with BM‐derived CLPs and/or ETPs (Min *et al*., 2004). Unfortunately, however, BM‐derived progenitor numbers decline markedly with age due to increased apoptosis rates as well as reduced proliferative capacities (Min *et al*., 2004). This in turn negatively impacts the delicate thymic stromal compartment, which is dependent on cross talk interactions with thymic progenitors for its sustained survival and morphogenesis (Shores *et al*., 1991; Hollander *et al*., 1995; van Ewijk *et al*., 2000; Dudakov *et al*., 2012). In this regard, two major points can be concluded based on observations made in rIL‐21‐treated aged mice. First, a report by Ozaki *et al*. previously demonstrated that IL‐21 overexpression in WT young mice expands LSK cells *in vivo* (Ozaki *et al*., 2006). Such effect is certainly beneficial as it can increase the pool of BM progenitor cells available for thymic migration. Although we did not directly assess the level of thymus‐migrating BM progenitors, we confirmed that all LSK subpopulations in ageing mice express IL‐21R but fail to proliferate in response to rIL‐21 administration (Fig. S2). Such apparent discrepancy between our observations and those reported by Ozaki and colleagues may be explained by the lower dose or bioavailability of rIL‐21 in comparison with *in vivo* overexpression. However, age‐related changes in the functional properties of LSK cells should also be taken into account. While the frequency of LT‐HSCs increases drastically in the BM of ageing mice, a decrease in the ST‐HSC and the transiently reconstituting MPP populations occurs in parallel (confirmed in Fig. S2) (Rossi *et al*., 2005). Such alterations are elemental to thymopoiesis as the ST‐HSC and MPP fractions both lie upstream of CLP and contribute to lymphoid reconstitution (Rossi *et al*., 2005). These observations are consistent with microarray analyses revealing the existence of systematic downregulation of LT‐HSC‐specific genes mediating lymphoid specification and function with ageing (Rossi *et al*., 2005). This implies that rIL‐21 can compensate for deficiencies affecting BM‐derived progenitors by stimulating the expansion of progenitor cells such as ETPs that have already seeded the thymic compartment. Second, the increase in the absolute number of cTEC and mTEC subpopulations in rIL‐21‐treated aged mice cannot be mediated by a direct action of rIL‐21 due to the absence of IL‐21R on TEC surface (Rafei *et al*., 2013a). The simplest interpretation of these results is that increased thymocyte numbers enhanced lympho‐stromal interactions consequently promoting TECs expansion (Hollander *et al*., 1995; van Ewijk *et al*., 2000). Indeed, BM transplantation studies using RAG^null^ mice as recipients revealed a central role played by developing thymocytes in the functional organization of thymic microenvironments (van Ewijk *et al*., 2000). By first providing signals to cTECs, DN thymocytes initiate the creation of a functional 3D organized cortical microenvironment. Such restructuring facilitates the differentiation of DN thymocytes to DP and SP stages, which ends up improving the survival/expansion of the mTEC compartment (van Ewijk *et al*., 2000). This is certainly a plausible explanation as the adult thymus contains nonsenescent progenitor stromal cells believed to be involved in TEC maintenance (Dumont‐Lagace *et al*., 2014). Thus, an involuted stroma possibly retains the capacity to regenerate in response to increased progenitor numbers. Under such context, the lack of rIL‐21‐induced thymopoiesis in younger mice may not be surprising as both thymic size and function are optimal at that age.

The concept that miR‐181a acts as a rheostat for TCR signaling is not novel (Li *et al*., 2007). Although expression of signaling molecules involved in TCR signaling is not influenced by age (Tamura *et al*., 2000), cumulative homeostatic proliferation is believed to promote progressive loss of miR‐181a in ageing naïve T cells overtime (Li *et al*., 2012). Only a continuous stream of newly developed T cells capable of replenishing the peripheral T‐cell compartment can compensate for such intrinsic defect. In accordance with these studies, we detected lower expression of miR‐181a in T cells of aged mice compared to their younger counterparts, which was further reflected on their poor TCR responsiveness (Fig. 4). However, *in vivo* provision of exogenous rIL‐21 reversed the miR‐181a decrease through enhanced *de novo* generation of RTEs that have incorporated the peripheral compartment. RTE maturation thereafter led to an increase in mature naïve T cells, which were competent enough to effectively respond to Trp2 vaccination and trigger a substantial delay in B16 tumor growth (Figs 3, 5, and 6). This, however, does not preclude the possibility that rIL‐21 administration to aged mice affects other preexisting immune cells in periphery. Additional studies involving thymectomized mice or adoptive transfer into athymic nude are required to confirm this hypothesis. In addition, there are data indicating that RTEs generated in aged hosts retain some intrinsic defects following TCR cross‐linking (Clise‐Dwyer *et al*., 2007). If so, how can we interpret the improved effector functions observed in the rejuvenated T‐cell pool of rIL‐21‐treated mice? Clise‐Dwyer *et al*. demonstrated that RTEs generated either following transplantation of younger hosts with aged BM or after antibody‐mediated depletion of peripheral T‐cell in ageing hosts exhibit normal effector function in comparison with RTEs developed in untreated aged animals (Clise‐Dwyer *et al*., 2007). The authors explained this conundrum by suggesting that under lymphopenic conditions, levels of IL‐7 are either elevated or competition for IL‐7 is reduced resulting in rescued T‐cell development. As stromal cells are the main producers of IL‐7 in the thymus, an ageing thymic microenvironment with increased TECs turnover would indeed compromise RTEs number, quality, and function due to limited IL‐7 availability. This highlights one of the distinctive thymopoiesis‐stimulating properties of rIL‐21 as it can raise the level of intrathymic IL‐7 production (Fig. 1SE) most likely by indirectly mediating the expansion of the stromal compartment through enhanced thymocyte proliferation therefore resulting in the generation of defects‐free RTEs.

Besides physiological ageing, contraction of the TCR repertoire is commonly observed in patients suffering from infections, cancers, or following BM transplantation (van den Brink *et al*., 2004). There are currently no effective therapies capable of exerting a positive impact on broadening the spectrum of TCR. Studies involving IL‐21R^−/−^ mice clearly showed that IL‐21 is dispensable for immune cell development as normal proportions of lymphocytes, monocytes, and granulocytes have been reported (Spolski & Leonard, 2014). Our data nevertheless suggest that rIL‐21 administration to ageing hosts could have potent clinical uses related to its ability to promote the expansion of thymic progenitor cells, which can be further enhanced if combined with other thymostimulatory compounds.

## Experimental procedures

### Cell line and mice

The B16F0 mouse melanoma cell line was kindly provided by Dr. J. Galipeau (Atlanta, GA, USA). The RAG2p‐GFP transgenic mice were kindly provided by Dr. M. Nussenzweig (Rockefeller University, New York, NY, USA). Female WT C57BL/6 mice were purchased from the Jackson Laboratory (Bar Harbor, ME, USA). To generate IL‐21R^−/−^ C57BL/6 mice, commercially available sperm was purchased from the MMRRC repository and used to fertilize female WT C57BL/6 mice. All mice were housed at the Institute for Research in Immunology and Cancer (IRIC) animal facility under specific pathogen‐free conditions. All animal protocols were approved by the Animal Care Committee of Université de Montréal.

### Antibodies, cytokines and reagents

Mouse rIL‐21 and granulocyte–macrophage colony‐stimulating factor (rGM‐CSF) were purchased from Peprotech (Rocky Hill, NJ, USA). All flow cytometry antibodies and Cytofix/Cytoperm Kits were purchased from BD Pharmingen (San Diego, CA, USA). The anti‐SHP‐2, PTPN‐22, DUSP5/6, and β‐actin antibodies used in Western blotting were purchased from Abcam (Cambridge, MA, USA). Quantikines were purchased from R&D System (Minneapolis, MN, USA). The CD3‐CD28 beads, cell trace, and Trizol were purchased from Invitrogen (Burlington, ON, Canada). The cell Lytic M buffer and lipopolysaccharide (LPS) were purchased from Sigma (St‐Louis, MO, USA). T‐cell enrichment kits were purchased from StemCell Technologies (Vancouver, BC, Canada). Peptides were synthesized by GenScript (Piscataway, NJ, USA).

### Administration of rIL‐21 and T‐cell analyses

For IL‐21 dosage establishment, WT young (2M), or aged (15M) female mice were IP‐injected with rIL‐21 in 200 μL PBS on a daily interval (total of five injections) in the first week. Control mice received equal volumes of PBS. All subsequent *in vivo* experiments were performed using rIL‐21 at 50 μg kg^−1^. Peripheral T‐cell absolute numbers were obtained by combining the analysis of blood samples collected from treated RAG2p‐GFP mice using the Scil‐Vet ABC^+^ hematological analyzer (to obtain absolute counts of lymphocytes) and flow cytometry (to obtain percentages of specific subpopulations).

### Enrichment of primary TECs

Primary TECs were enriched from thymic lobes of WT C57Bl/6 mice as previously described (Gray *et al*., 2006).

### Western blots

Splenocytes were first isolated to sort total CD3^+^ T cells. Whole T‐cell extracts were obtained through using the Cell Lytic M reagent (Sigma). Extracts were separated by electrophoresis, transferred onto Hybond‐ECL membrane, and probed using primary antibodies. Densitometry analysis was conducted by first imaging the chemiluminescent blots with the ImageQuant LAS 4000 imager (GE Healthcare Life Sciences, Mississauga, ON, Canada) followed by analysis using the ImageQuant TL software (GE Healthcare Life Sciences).

### Expression analysis of miR‐181a

T cells were first sorted directly in 900 μL Trizol (10^6^ cells per tube) and lysed followed by RNA extraction in Trizol reagent (Invitrogen). RNA purification was further carried out using the RNA extraction kit (QIAgen, Toronto, On, Canada). Reverse transcription was performed using the High Capacity cDNA reverse transcription kit and qPCR was performed with a 7900HT Fast Real‐Time PCR system at IRIC's genomic core facility. Target gene values were normalized to endogenous control *Gapdh*.

### Intracellular staining

For analysis of phosphorylated (p)Lck, ZAP‐70, and ERK, isolated splenocytes were first stimulated *in vitro* using CD3‐CD28 Dynabeads (Invitrogen) for 5 min, then washed and surface stained for CD4 and CD8 prior to intracellular staining according to manufacturer's instructions.

### Histological analyses

Chosen organs were harvested from treated mice, fixed in 10% formalin before mounting in paraffin. Sections were then stained with hematoxylin and eosin, then scanned using the NanoZoomer Digital Pathology system and NPD.scan 2.3.4 software (Hamamatsu, Hamamatsu city, Japan).

### DC vaccination and tumor challenge

To generate DCs for vaccination, BM cells were extracted from tibia/femur bones of 8‐week‐old male mice and plated in 10‐cm nontissue culture‐treated Petri dishes containing 10 mL of complete RPMI 1640 medium (0.048 mmol L^−1^ β‐mercaptoethanol, 2 mmol L^−1^ l‐glutamine, 10% fetal bovine serum, 2 mmol L^−1^ penicillin–streptavidin) supplemented with 10 ng mL^−1^ rGM‐CSF (Peprotech). The media were changed at days 3 and 6 of culture. To induce DC maturation, 1 μg mL^−1^ LPS (Sigma) was added at day 8 for 24 h. Following confirmation of a mature phenotype (>80% of adherent cells were CD80^+^ CD86^+^ MHCI^+^ MHCII^+^), DCs were pulsed with OVA‐ or Trp2‐derived peptides (GenScript) for 4 h, then harvested for intravenous vaccination. Two weeks following the last boost, mice were SC‐challenged with 5 × 10^5^ B16 tumor cells. Tumor volume was measured as [(length × width^2^)/2], and mice were sacrificed when the tumor volume reached 2000 mm^3^.

### Proliferation and cytokine measurements

For all assays conducted using nonvaccinated mice, total or fractionated T cells were first isolated by negative selection, then plated at 10^5^ cells well^−1^ in 96‐well plates. To trigger proliferation/activation, T cells were stimulated with CD3‐CD28 Dynabeads in a 1:1 ratio. For *in vitro* recall responses following vaccination, splenocytes were pulsed with 1 μg mL^−1^ Trp2 peptide. Cells or supernatants were collected 48 h poststimulation for further analyses.

### Statistical analyses

*P*‐values were calculated using the ANOVA and log‐rank statistical test where applicable. Log‐rank testing was performed using software available at the Walter and Eliza Hall Institute Web site (http://bioinf.wehi.edu.au/software/russell/logrank/).

## Author contributions

EAC designed most of the study, carried out experiments, analyzed the data, prepared the figures, and wrote the first draft of the manuscript. AT, SP, RK, and SZ performed some experiments and contributed to data analysis and manuscript preparation. MR designed the study, discussed the results with all authors, and wrote the manuscript. The authors declare that they have no conflict of interest.

## Supporting information


**Fig. S1** Analysis of thymic GFP^+^ content, absolute counts and IL‐7 production following rIL‐21 administration.
**Fig. S2** Administration of rIL‐21 to aged mice has no beneficial effect on the BM compartment.
**Fig. S3** Assessment of TCR repertoire diversity following rIL‐21 administration to young mice.
**Fig. S4** Transcription factors quantification in T cells derived from treated mice.
**Fig. S5** Assessment of autoimmune signs following rIL‐21 administration.
**Fig. S6** Generation and characterization of BM‐derived DC for vaccination.
**Fig. S7** rIL‐21 administration to aged mice increases thymic output leading to improved immune functions.
