# Supplementary material for: Interleukin‐21 administration to aged mice rejuvenates their peripheral T‐cell pool by triggering de novo thymopoiesis
Source: Aging Cell. 2016 Jan 13;15(2):349–60. doi: 10.1111/acel.12440 (PMC4783337; doi:10.1111/acel.12440)
Supplement: Supplementary file 1 — Fig. S1 Analysis of thymic GFP+ content, absolute counts and IL‐7 production following rIL‐21 administration. Fig. S2 Administration of rIL‐21 to aged mice has no beneficial effect on the BM compartment. Fig. S3 Assessment of TCR repertoire diversity following rIL‐21 administration to young mice. Fig. S4 Transcription factors quantification in T cells derived from treated mice. Fig. S5 Assessment of autoimmune signs following rIL‐21 administration. Fig. S6 Generation and characterization of BM‐derived DC for vaccination. Fig. S7 rIL‐21 administration to aged mice increases thymic output leading to improved immune functions. [file ACEL-15-349-s001.docx]

**Supplementary Figure S1:** **Analysis of thymic GFP^+^ content, absolute counts and IL-7 production following rIL-21 administration.**

A) Schematic diagram representing the strategy and group of mice used to identify the optimal dose of rIL-21 capable of enhancing *de novo* thymopoiesis. Thymi were collected and analyzed one week following the last rIL-21 administration. B) Total thymocyte count following administration of ascending doses of rIL-21 to young (2M) or aged (15M) WT female C57BL/6 mice. C) Representative flow-cytometry analysis of thymic GFP content. D) Absolute counts of total thymocytes in 2M (PBS ) or 15M (PBS ) 1 week post-PBS treatment versus 15M (rIL-21

) at 1, 2, and 3 weeks post-rIL-21 treatment. E) ELISA-based quantification of IL-7 secretion by thymus one week post-treatment. All data are representative of three independent experiments (n = 5/group with *p˂0.05).

**Supplementary Figure S2: Administration of rIL-21 to aged mice has no beneficial effect on the BM compartment.**

A) Representative flow-cytometry analysis of IL-21R expression on the surface of LT-, ST-HSCs and MPPs derived from WT (top panels) or IL-21R^-/-^ mice (lower panels). Filled histograms represent IL-21R expression. B-C) Percentages of total LSK cells or LT-, ST-HSCs as well as MPPs as depicted by flow-cytometry. D) Absolute counts of LSK sub-populations in 2M (PBS ), 15M (PBS ), and 15M (rIL-21 ) aged mice. E) CLP percentage and absolute counts in 2M (PBS ), 15M (PBS ), and 15M (rIL-21 ) aged mice. F) Absolute counts of LSK derived from young or aged mice following *in vitro* PBS or rIL-21 treatment. All data are representative of three independent experiments (n=5/group with *p˂0.05; **p˂0.01, and ***p˂0.001).

**Supplementary figure S3. Assessment of TCR repertoire diversity following rIL-21 administration to young mice**.

Flow-cytometry analysis of 15 TCRVβ-chains using spleen-derived CD4^+^ (top panel) or CD8^+^ (lower panel) T cells from young 2M old mice treated with PBS (black bars) or rIL-21 (white bard). No significant differences were depicted between both groups. All data are representative of three independent experiments (n = 5/group).

**Supplementary Figure S4:** **Transcription factors quantification in T cells derived from treated mice.**

T cells derived from treated mice were isolated then lysed to extract total mRNA. qPCR analyses were then conducted to quantify the expression of targeted genes relative to endogenous controls. Groups are displayed as: 2M (PBS ); 15M (PBS ); and 15M (rIL-21 ) aged mice. All data are representative of three independent experiments (n=5/group with *p˂0.05, ***p˂0.001, and ****p˂0.0001).

**Supplementary Figure S5: Assessment of autoimmune signs following rIL-21 administration.**

A) A representative photograph of spleens and their relative spleen to body weight derived from treated mice. B) Serum IgG levels quantified by ELISA. C) Hematoxylin-eosin staining of formalin-fixed sections of lung, liver and kidney of young, PBS- or rIL-21-treated aged mice. Scale bars represent 2 mm. D) Representative flow-cytometry analysis of CD4^+^CD25^+^ T_regs_ in all experimental groups. We pre-gated on CD4^+^ T cells prior to CD25^+^ (cell surface) and FOXP3 (intracellular) analysis. E) Quantification of CD4^+^CD25^+^ T_regs_ according to flow-cytometry analysis and splenocyte counts. All data are representative of three independent experiments (n = 5/group with ***p˂0.001).

**Supplementary Figure S6:** **Generation and characterisation of BM-derived DC for vaccination.**

A) To generate BM-derived DCs, femur and tibias of male C57BL/6 mice where flushed to collect total nucleated cells, which were then plated for 8 days with rGM-CSF (10ng/ml). LPS was added to stimulate DC maturation at day 9. B) Representative flow-cytometry analysis of mature DC phenotype (>80% of DCs were CD11c^+^CD80^+^CD86^+^H2-K^b+^I-A^b+^) using all non-adherent cells collected at day 10.

**Supplementary Figure S7: rIL-21 administration to aged mice increases thymic output leading to improved immune functions.**

In aging mice, thymopoiesis is diminished causing attrition of the TCR repertoire and accumulation of peripheral CD44^hi^ T cells. In parallel, increased post-thymic life span leads to accumulated SHP-2 and DUSP5/6 due to decreased expression of miR-181a. As such, TCR stimulation of aging naïve T cells is weakened leading to lower CD25 expression along with poor secretion of IL-2 and thus, impaired immune functions. When given to aged mice, rIL-21 promotes thymopoiesis leading to increased T-cell output and improved TCR diversity. As RTEs increase the proportion of naïve T cells expressing higher level of miR-181a, SHP-2 and DUSP5/6 levels diminish allowing better T-cell responses translating into potent immunity.
